# Contrastive learning for passive acoustic monitoring: A framework for sound source discovery and cross-site comparison in marine soundscapes

**DOI:** 10.1371/journal.pcbi.1014005

**Published:** 2026-03-06

**Authors:** Richard Acs, Ali Ibrahim, Hanqi Zhuang, Laurent M. Chérubin

**Affiliations:** 1 Department of Electrical Engineering and Computer Science, Florida Atlantic University, Boca Raton, Florida, United States of America; 2 Harbor Branch Oceanographic Institute, Florida Atlantic University, Fort Pierce, Florida, United States of America; Tilburg University Faculty Humanities: Tilburg University Tilburg School of Humanities and Digital Sciences, NETHERLANDS, KINGDOM OF THE

## Abstract

Passive acoustic monitoring (PAM) is a powerful tool for studying marine biodiversity, but large-scale analysis of underwater recordings is constrained by noise, overlapping signals, and limited labeled data. Here, we present a scalable, unsupervised contrastive learning framework for marine soundscapes. Using a large PAM dataset spanning multiple biogeographies, we show that the proposed approach organizes recordings into clusters with well-defined internal structure, as assessed using intrinsic clustering metrics and within-cluster similarity. The resulting clusters reveal recurring acoustic patterns that correspond to broad sound-source categories, including biological sounds such as fish calls and choruses, and anthropogenic sounds such as vessel noise, without explicitly enforcing these distinctions during training. Compared with established approaches, including cepstral features, variational autoencoders, and supervised pipelines, the proposed framework produces embeddings that support more compact and stable unsupervised clustering while preserving fine-scale acoustic variation beyond predefined species labels. By learning a shared representation across recordings from multiple sites and years, we examine the reproducibility of acoustic patterns across locations and identify both site-shared and site-specific sound signatures. Although the method is not designed to recover coarse species labels, it enables label-efficient analysis by reducing reliance on manual annotation and supporting exploratory characterization of complex marine soundscapes. Together, these results highlight multi-positive contrastive learning with a teacher network and acoustically informed augmentations as an effective strategy for scalable, discovery-driven analysis of passive acoustic monitoring data.

## Author summary

Underwater ecosystems are rich with sound, ranging from fish calls and choruses to the noise generated by human activity, yet large-scale analysis of passive acoustic recordings is hindered by background noise, overlapping signals, and sparse annotation. We present a self-supervised machine learning framework that learns directly from reef acoustic recordings without requiring manual labels. Using contrastive learning, the model organizes sound fragments based on acoustic similarity, enabling unsupervised structuring of thousands of hours of audio. Applied to recordings from multiple Caribbean spawning aggregation sites, the approach revealed recurring acoustic patterns consistent with known fish vocalization activity, as well as site-specific sound types and distinct anthropogenic noise signatures. Compared with conventional feature-based and supervised methods, the proposed framework produces more stable and coherent acoustic groupings while remaining entirely label-free. These results illustrate how self-supervised learning can support scalable, data-driven exploration of passive acoustic monitoring data for characterizing marine soundscapes.

## Introduction

Large-scale acoustic datasets pose fundamental challenges for machine learning: they are high-dimensional, noisy, and often sparsely labeled. Overlapping sources, variable environments, and low signal-to-noise ratios complicate the extraction of meaningful patterns [1,2]. Supervised approaches are further limited by their reliance on costly expert annotations, which are rarely available at scale [3]. In Passive Acoustic Monitoring (PAM), these challenges are compounded by repeated but non-identical events and continuous variation in call structure, complicating the definition of discrete classes and similarity relationships [4]. Representation-learning methods must therefore contend with continuous acoustic manifolds, overlapping sources, and incomplete or noisy labels.

PAM enables non-invasive, long-term observation of soniferous species across large and often inaccessible environments [4]. In tropical and subtropical marine systems, acoustic recordings have been used to characterize fish communities [4,5], identify spawning aggregations [5,6], and detect species-specific sonic behaviors [5,7,8]. Spawning aggregation sites, where large numbers of fish gather seasonally for reproduction, often produce dense choruses that dominate the local soundscape [5,6]. Despite substantial advances, extracting consistent and generalizable insights from large-scale PAM data remains challenging due to strong spatiotemporal variability and sparse or incomplete annotation [9].

Supervised methods perform well in classification tasks but are inherently constrained by predefined classes and label availability [4]. PAM increasingly demands representation-learning approaches that organize acoustic sources without assuming fixed taxonomies [9]. Self-supervised learning enables models to capture structure at multiple levels of granularity, supporting discovery-driven analyses and cross-site comparisons beyond a limited set of annotated classes [10]. However, biologically meaningful sounds often recur without being identical, forming continuous manifolds rather than discrete categories. Much PAM data remains unlabeled or ambiguously labeled, and overlapping biological and anthropogenic sources further blur category boundaries [11]. Unsupervised and self-supervised methods must therefore balance two competing risks, over-separating acoustically related events into fragmented clusters, or collapsing distinct but related call types into overly coarse representations. These challenges complicate both clustering-based discovery and ecological interpretation.

Early bioacoustic analysis relied on handcrafted spectral features and ecoacoustic indices to summarize soundscape structure [12,13]. Classical machine learning methods applied to features such as MFCCs (Mel-Frequency Cepstral Coefficients) and GTCCs (Gammatone Cepstral Coefficients) achieved strong performance on small, curated datasets [5,14], but required extensive feature engineering and degraded in large-scale, noisy PAM settings [4,11]. Deep Convolutional Neural Networks (CNNs) improved performance by learning spectro-temporal representations directly from data, outperforming handcrafted pipelines in detection and classification tasks [2,15]. Pretrained CNN embeddings further enabled large-scale ecological assessments [16]. However, CNN-based approaches remain fundamentally label-driven and may collapse meaningful acoustic variation within predefined classes, limiting their suitability for unsupervised discovery.

Beyond CNNs, several unsupervised representation-learning approaches have been explored. Variational Autoencoders (VAEs), Self-Organizing Maps (SOMs), and Gaussian Mixture Models (GMMs) have been used to uncover latent acoustic structure and cluster embeddings into interpretable units [17,18]. Extensions such as the Gaussian Mixture VAE (GMVAE) integrate clustering directly into the latent space [18,19]. In marine PAM, autoencoder-based pipelines and Autoencoder–HDBSCAN frameworks have shown promise on curated, high-SNR datasets [20,21]. More recently, transformer-based audio models have been explored for representation learning [22]. However, these approaches typically rely on large curated corpora and substantial computational resources, limiting their applicability to noisy, continuous reef soundscapes. Across these methods, reconstruction-driven or architecture-centric objectives remain poorly matched to environments characterized by overlapping events, low SNR, and continuous acoustic variation.

Contrastive learning offers a promising alternative by enforcing structure in the representation space through positive and negative pairs constructed via augmentations or labels. Methods such as SimCLR [23] and SupCon [24] improve intra-class cohesion and inter-class separation by encouraging positive pairs to remain close while separating them from other samples in the batch. However, standard contrastive objectives assume well-defined positives and discrete class boundaries—assumptions frequently violated in bioacoustic data. Acoustically related but non-identical events may be pushed apart, while overly broad positives can collapse distinct call types. Despite these limitations, contrastive learning reduces annotation dependence and improves representation stability compared to purely unsupervised clustering pipelines such as VAE + GMM. While promising results have emerged in bioacoustics [10,25], applications to large-scale, multi-site PAM remain scarce.

In this study, we address these gaps by introducing a marine PAM–adapted contrastive learning framework based on SimCLR. We assemble a large cross-site, multi-year datasets of marine spawning aggregation site acoustic recordings in the Caribbean, incorporating both labeled and unlabeled data. To enable within-species call type analysis, we leverage labels generated by a pretrained CNN classifier developed mostly on Caribbean PAM data [26], covering six acoustic classes: red hind (*Epinephelus guttatus*), Nassau Grouper (*Epinephelus striatus*), black grouper (*Mycteroperca bonaci*), yellowfin grouper (*Mycteroperca venenosa*), squirrelfish (holocentridae), and vessel/anthropogenic sounds. This setup allows us to systematically evaluate cross-site composition while probing species-level and call types structure.

We benchmark our approach against several families of baselines, including a VAE + GMM pipeline for unsupervised call types clustering [17], a supervised contrastive SimCLR variant that incorporates species labels during training [24], and classical GTCC and MFCC feature-based clustering methods [4,14].

Our contributions are threefold:

1. We analyze a large, multi-site marine PAM dataset from Caribbean spawning aggregation sites collected over multiple years to evaluate unsupervised representation learning under realistic, noisy reef conditions.
2. We propose a domain-adapted variant of SimCLR for marine PAM that incorporates a teacher network, multi-positive contrastive objectives, stability regularization, and acoustically appropriate augmentations to enable fine-grained sound source discovery and inter-site comparisons.
3. We provide a systematic benchmark of unsupervised and semi-supervised pipelines, showing that our unsupervised clustering approach yields stable internal structure across sites and supports exploratory analysis of biological and anthropogenic sound patterns.

Together, this work positions passive acoustic monitoring not only as a conservation and biodiversity monitoring tool but also as a challenging and impactful benchmark to advance scalable machine learning methods capable of handling real-world acoustic complexity.

## Materials and methods

To establish a pipeline that most effectively encodes PAM data for subsequent clustering and unique call signature detection, we evaluated a range of feature extraction, dimensionality reduction, and clustering techniques. The subsequent sections first provide an overview of the experimental focus. Then, we describe our data and preprocessing techniques, feature extraction methods, clustering methods, and the overall experiments and performance metrics that we conducted.

### Experimental overview

**Fig 1:**
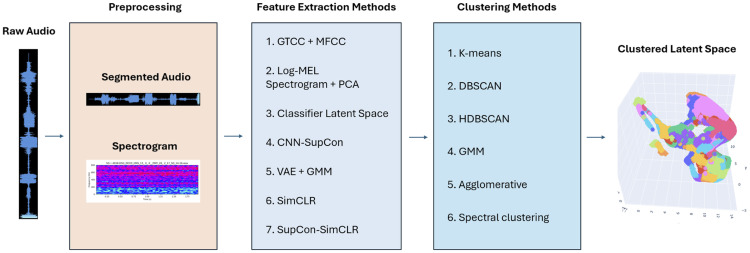
Overview of the experimental workflow. Three evaluation modes were applied: (1) label agreement with FADAR classes, (2) unsupervised cluster quality, and (3) acoustic pattern discovery.

This section provides an overview of the experimental framework used to evaluate acoustic representations derived from multi-site passive acoustic monitoring (PAM) recordings. All experiments follow a common workflow (Fig 1). Raw recordings are preprocessed, segmented into short time windows, and transformed into acoustic embeddings using the feature extraction or representation learning methods detailed below. These embeddings are subsequently clustered and evaluated using complementary quantitative and qualitative metrics. The embedding methods first describe baselines, followed by our PAM-adapted self supervised contrastive learning framework.

The evaluation is organized into three experiments. Experiment 1 measures agreement with coarse ecological labels. Experiment 2 examines intrinsic cluster structure independent of labels. Experiment 3 assesses whether representations support the discovery of reproducible acoustic signatures across geographically distinct sites.

### Dataset and preprocessing

We compiled a multi-site dataset of underwater acoustic recordings collected from acoustic recorders deployed at seven spawning aggregation sites in the Caribbean region between 2017 and 2024. Three of these sites are located in the coastal waters of Mexico (Xcalak, San Juan, and Punta Allen) in the western Caribbean. The other sites are located in the Mona Passage, west of Puerto Rico (Abrir la Sierra (ALS; ALS Deep; Bajo de Sico (BDS); Mona Elbow; Mona H6) and in St. Thomas, US Virgin Islands (Red Hind Bank (RHB) and Grammanik Bank (GB)), both islands being located in the northern Caribbean. The sites in Mexico are known Nassau grouper spawning aggregation sites [27]. The ALS site is a known red hind only spawning aggregation site, while ALS Deep and BDS are known Nassau grouper spawning sites [28]. The sites near Mona Island are known as multi-species (Elbow) and Yellowfin grouper (H6) spawning sites [29]. GB is a known yellowfin and Nassau grouper spawning aggregation site [30,31] and RHB is a known red hind only spawning site [32]. The recorders were programmed to record 20-second audio segments at five-minute intervals over 3–6 month periods. The dominant species at each site were identified by expert audit and automated classification using the Fish Acoustic Detection Research Algorithm (FADAR) [26].

FADAR was applied to all recordings, generating soft-label predictions for 2-second segments across six categories: (1) Red Hind, (2) Black Grouper, (3) Yellowfin Grouper, (4) Nassau Grouper, (5) Squirrelfish, and (6) Vessel/Other sounds. Segments classified as “noise” were excluded. To prevent class imbalance across sites and years, we employed stratified random sampling such that each class contained the same number of samples as the smallest class (Squirrelfish). The final dataset contained 413,272 labeled segments (≈70,000  per class) distributed across all sites and years shown in Table 1. This specific dataset was used to and train the method proposed in this study. The unsupervised clustering algorithm was then applied to the entire dataset one location at a time for analysis, which in addition to those listed in Table 1 includes Punta Allen, San Juan, and Mona H6.

**Table 1:** Passive acoustic training data site locations and species. The number of recordings represents the number of 20-second records received from recorders for each site before pre-processing and balancing.

Location	Year	Number of recordings	Dominant species
Mexico, Xcalak	2024, 2022	24,295	Nassau Grouper
Puerto Rico, ALS	2022	57,315	Red Hind
Puerto Rico, ALS Deep	2022	65,084	Nassau Grouper
Puerto Rico, Mona Elbow	2017	59,641	Multispecies
Puerto Rico, BDS	2017	85,987	Nassau Grouper
Saint Thomas, GB	2017	97,621	Yellowfin/Nassau Grouper
Saint Thomas, RHB	2017	43,547	Red Hind

**Fig 2:**
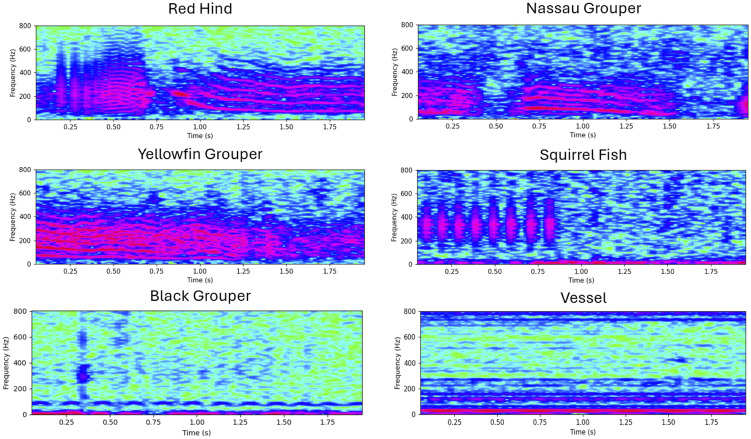
Example spectrograms for each of the six FADAR-defined classes. Call samples are given from the labeled dataset examples used to train FADAR [26], and reflect the model structure for each sound type.

We include examples of each class (see Fig 2).

Preprocessing. All recordings were downsampled to 10 kHz from native 44.1 kHz to reduce storage and computation while preserving the relevant frequency range of fish calls and vessel noise (0–5 kHz). Each 20-second clip was segmented into non-overlapping 2-second windows. For deep learning approaches (SimCLR, PAM-SimCLR SupCon SimCLR, VAE + GMM), each waveform was converted into a log-MEL spectrogram using: (1) Short-Time Fourier Transform (STFT) with a 25 ms Hamming window and 10 ms hop length, (2) projection to 128 MEL-spaced frequency bins (0–5 kHz), (3) log compression log(1+x), and (4) normalization to zero mean and unit variance. Although MEL scaling is human-perception–based, it remains standard in bioacoustic workflows. To prevent data leakage across partitions, we performed an 80/20 split at the level of the original 20-second recordings prior to segmentation. All 2-second windows derived from a given recording were assigned exclusively to either the training or testing set. The held-out test set was used to compute the clustering evaluation metrics reported in Table 5 and Fig 5. For the call signature discovery analysis, we embedded and clustered the full dataset (training + testing), but results were examined separately for each site to ensure that all acoustically relevant patterns present in the datasets were included subsequent interpretation.

**Fig 3:**
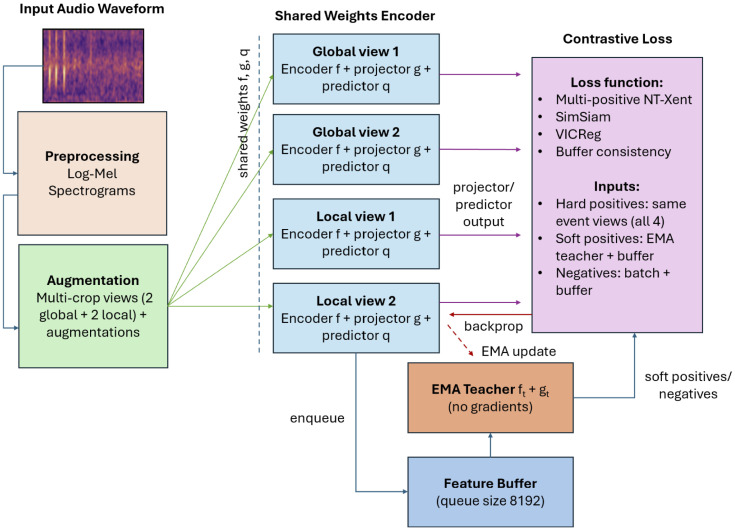
Architecture of the proposed PAM-SimCLR framework. Multiple augmented views (two global and local crops) are processed by a shared ResNet-18 encoder and projection head, with an EMA teacher providing soft multi-positive/negative targets.

**Fig 4:**
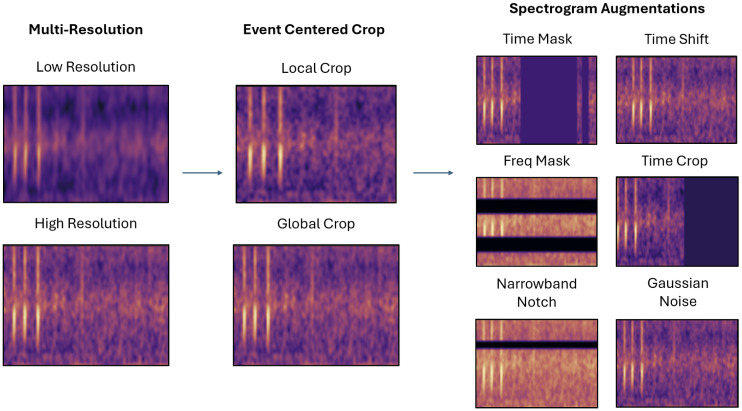
Augmentations used to create positive pairs. Each spectrogram is cropped globally or locally and then transformed by time/frequency masking, spectral notching, temporal shift/truncation, or Gaussian noise.

### Feature extraction approaches

To evaluate the impact of representation choice on clustering performance, we compared classical acoustic features, generative models, and contrastive learning variants against our proposed PAM-adapted SimCLR framework. Table 2 provides a concise overview.

**Table 2:** Summary of feature extraction methods. Full implementation details are provided in S2 Appendix.

Method	Type	Dimensionality	Clustering
GTCC+MFCC	Cepstral	93	KMeans
Log-Mel + PCA	Linear projection	100	KMeans
VAE + GMM	Generative latent space	64	GMM
PAM-SimCLR	Unsupervised contrastive embeddings	128	KMeans
Vanilla SimCLR	Unsupervised contrastive embeddings	128	KMeans
supCon-SimCLR	Semi-supervised contrastive embeddings	128	KMeans
CNN-SupCon	Sup. contrastive embeddings	128	KMeans

#### Classical features.

We extracted Mel-Frequency Cepstral Coefficients (MFCCs, 13 coefficients) and Gammatone Cepstral Coefficients (GTCCs, 80 coefficients), concatenating the two since preliminary tests showed improved performance over either alone [14]. We also generated log-Mel spectrograms (64 bands) and applied PCA, retaining the top 100 components. All features were implemented using librosa and SciPy.

**Generative baseline.** As an unsupervised generative model, we trained a VAE on log-Mel spectrograms, with embeddings clustered using a Gaussian Mixture Model (GMM). This follows prior work in acoustic unit discovery [17,18,20,21]. The VAE served as a baseline for comparison with our contrastive approach.

### Contrastive learning approaches

#### Self-supervised contrastive learning.

We propose a domain-adapted contrastive learning framework based on SimCLR that is designed to address key challenges of PAM data: repeated but non-identical acoustic events, overlapping sound sources, low signal-to-noise ratios, and sparse or imperfect labels. The proposed framework introduces three main components beyond standard SimCLR: (i) multi-scale event-centric views to capture both coarse call context and fine temporal–spectral detail; (ii) a teacher-guided multi-positive contrastive objective that allows acoustically similar but non-identical events to be treated as positives; and (iii) auxiliary regularization terms that stabilize training and prevent representation collapse. Each component is intended to preserve meaningful acoustic variability while maintaining separation between distinct sound types.

Our main contribution is a contrastive learning framework adapted from SimCLR and tailored to marine PAM (Fig 3). Each audio clip is rendered into multi-resolution log-Mel spectrograms, which serve as the input to the contrastive framework. Event-centric cropping generates both global and local views, which are augmented through a suite of operations reflecting reef noise conditions (time/frequency masking, spectral notches, temporal shifts, truncation, Gaussian noise), as illustrated in Fig 4.

Each spectrogram was transformed into four augmented views using a multi-scale cropping strategy. Two global crops, each consisting of a 256-frame window, were sampled at independent random offsets and augmented separately. Two local crops, each consisting of a 96-frame window, were centered on high-energy regions of the spectrogram and sampled with ±24-frame temporal jitter, with independent augmentations applied to each. Global crops preserve coarse call structure and background context, while local crops emphasize fine temporal–spectral details such as harmonics and pulses. This multi-scale design provides complementary views that enhance robustness to overlapping calls and background noise in reef soundscapes. All crops share a ResNet-18 backbone encoder, projected into a 128-D embedding space.

**Teacher-guided multi-positive contrastive learning** In standard SimCLR contrastive learning frameworks, every other sample in the batch is treated as a negative example. For passive acoustic monitoring, this assumption is problematic because different clips often contain distinct instances of the same call type (e.g., repeated pulses or whistles). Treating these naturally similar events as negatives forces the model to push them apart in the embedding space, fragmenting biologically meaningful structure and reducing cluster coherence. Our multi-positive formulation mitigates this issue by allowing the model to identify multiple acoustically similar calls as valid positives.

Each training sample is represented by four augmented spectrogram crops: two global views covering the full event context and two local views centered on higher-energy subsegments. The global views are used for the main contrastive loss, while the local views provide fine-scale invariance constraints through auxiliary objectives.

To address the variability of overlapping and repeated calls, we introduce a teacher-guided multi-positive contrastive loss. An exponential moving-average (EMA) teacher produces a soft similarity matrix that identifies both the paired augmentation and acoustically similar neighbors as positives. A FIFO feature bank of teacher embeddings from previous iterations further expands the pool of negatives, improving stability across training steps.

Formally, the contrastive objective extends the normalized temperature-scaled cross-entropy (NT-Xent) formulation as


$${ℒ}_{ctr}=-\frac{1}{2B}\sum_{i=1}^{2B}\sum_{j=1}^{2B+M}W_{ij}^{full}\;\log\frac{\exp(sim(z_{i},z_{j})/\tau )}{\sum_{l=1}^{2B+M}\exp(sim(z_{i},z_{l})/\tau )},\;$$ (1)

where all similarities are cosine similarities between online embeddings $z_{i}\;$ and $z_{j}\;$. Each batch provides *2B* global-view embeddings, and an additional *M* teacher embeddings from a FIFO feature bank (M=8192 ) contribute to the negative set.

The weighting matrix $W^{full}\;$ incorporates teacher-guided soft positives. Teacher embeddings $t_{i}\;$ and $t_{j}\;$ are compared using cosine similarity, and the top-*k* neighbors (k=5 ) whose similarity exceeds a fixed threshold (θ=0.7 ) are assigned fractional weights:


$$\begin{array}{c} W_{ij}=\left\{ \begin{array}{cc} 1, & \text{if }z_{j}\text{ is the paired global augmentation of }z_{i},\\ \frac{1+sim(t_{i},t_{j})}{2Z_{i}}, & \text{if }sim(t_{i},t_{j})>\theta \text{ and }j\in \text{top-}k,\\ 0, & \text{otherwise}, \end{array}\right.\; \end{array}$$

where $Z_{i}=\sum_{j}W_{ij}\;$ normalizes weights for each anchor. Self-pairs (i=j ) are masked. Teacher–teacher similarities $sim(t_{i},t_{j})$ are used for positive selection because the EMA teacher provides a more stable neighborhood estimate than the online encoder. This formulation enables each anchor to treat multiple acoustically similar calls as positives while preserving informative negative examples drawn from the batch and the feature bank.

This formulation allows the model to treat multiple acoustically similar calls as valid positives, preventing related events from being pushed apart and preserving diversity across distinct calls. The remaining two local views ($z_{\ell }\;$) are used for auxiliary objectives that promote stability and invariance: (i) a SimSiam-style term applied between a global and a local view, aligning each view‘s predictor output with a stop-gradient target from the other view, (ii) variance–invariance–covariance regularization (VICReg) that enforces non-collapse and decorrelation, and (iii) implicit feature-bank consistency via the slowly updated teacher queue. The overall training objective is a weighted combination of these components:


$$ℒ=\alpha \;{ℒ}_{ctr}+\beta \;{ℒ}_{siam}+\gamma \;{ℒ}_{vic},\;$$ (2)

where *α*, *β*, and *γ* balance the contributions of the contrastive, invariance, and regularization terms, respectively. Full mathematical definitions and derivations of each component are provided in S2 Appendix.

**Semi-supervised contrastive learning.** We also implemented a semi-supervised variant by incorporating FADAR-provided labels into the contrastive loss. In this formulation, positives are drawn from samples that share the same coarse class label, providing weak class-level guidance that encourages more compact intra-class clusters. This approach allows us to assess how limited annotation influences the learned representation, illustrating whether sparse labels (i) provide beneficial high-level structure or (ii) inadvertently distort the unsupervised organization of unlabeled call signatures.

**Fully-supervised contrastive learning.** As a supervised upper bound, we also trained a ResNet-18 encoder with supervised contrastive (SupCon) loss [24], which encourages intra-class compactness and inter-class separation. Labels are generated using classification output from FADAR [26]. Full training details are provided in Supplementary S2 Appendix.

**Vanilla SimCLR.** As a baseline, we implemented a vanilla SimCLR [23] model using a ResNet-18 backbone with a three-layer projection head, trained using the NT-Xent contrastive loss. To ensure a controlled ablation, vanilla SimCLR was trained using the same preprocessing, cropping strategy, augmentations, and optimization schedule as PAM-SimCLR. Specifically, two global views were generated per sample using event-centered crops computed from a base log-MEL representation, with temporal jitter applied during cropping. Both views were then augmented using the same spectrogram perturbations as our PAM-SimCLR. In contrast to PAM-SimCLR, vanilla SimCLR uses a single log-MEL configuration for all views, applies instance-level contrastive learning only, and does not incorporate multi-positive pairing, teacher guidance, or task-specific objectives.

### Clustering

For each feature extraction method, we benchmarked clustering performance using six algorithms spanning distinct paradigms: centroid-based (K-Means, GMM), hierarchical (Agglomerative), graph-based (Spectral), and density-based (DBSCAN, HDBSCAN) [33]. Methods requiring a fixed cluster count (K-Means, Agglomerative, Spectral, GMM) were set to k=6 , matching the six FADAR labels, while density-based approaches adaptively inferred structure from neighborhood and density criteria, capturing subclusters and outliers without preset *k*.

### Evaluation design and metrics

To assess the quality of different feature extraction methods, we used three complementary evaluation strategies: (a) how well unsupervised clusters aligned with known ecological classes (FADAR labels); (b) the intrinsic quality of clusters independent of labels, focusing on their compactness and separation; and (c) whether the framework could uncover reproducible acoustic signatures across geographic regions. Together, these experiments provided external, internal, and acoustic-pattern perspectives on representation quality.

Experiment 1: Label agreement. Clusters were compared to the six FADAR-defined classes using Adjusted Rand Index (ARI), Adjusted Mutual Information (AMI), and Hungarian Accuracy. FADAR achieves species-level sensitivity ranging from 0.91–1.00, specificity of approximately 0.99 across classes, and an overall accuracy of 97.5% on a manually annotated Caribbean dataset [26], indicating that these coarse labels are reliable for evaluating high-level agreement. Because FADAR does not distinguish among different call types or call variants within a species, some disagreement with unsupervised clusters is expected. ARI, AMI, and Hungarian Accuracy quantify chance-corrected agreement, shared mutual information, and optimal one-to-one matching accuracy, respectively. For this experiment, the number of clusters was fixed at six to match the six FADAR categories.

Experiment 2: Unsupervised cluster quality. To measure structure without reference to labels, we fixed the number of clusters at k=60  and applied Silhouette, Davies–Bouldin Index (DBI), and Calinski–Harabasz (CH) scores. These metrics quantify cohesion, separation, and cluster distinctness. Preliminary expert review suggested that k=60  best reflected meaningful sound sources granularity, but we also include a brief exploration of cluster number on silhouette score. Importantly, these metrics are interpreted relative to the marine PAM problem and serve to compare methods against one another, rather than as absolute measures of success or failure. To provide a reference point, we also evaluated our framework on two well-known acoustic benchmark datasets under cleaner conditions, establishing expected performance prior to application on the more challenging soundscape data. Table 3 shows the characteristics of the evaluation metrics utilized in both experiments 1 and 2.

**Table 3:** Evaluation metrics used in Experiments 1 and 2. Arrows indicate the desired direction of each score. Formal definitions are provided in S2 Appendix.

Metric	Purpose	Better	Range
Adjusted Rand Index (ARI)	Chance-corrected agreement with labels	Higher (↑)	[-1, 1]
Adjusted Mutual Information (AMI)	Shared information with labels	Higher (↑)	[0, 1]
Hungarian Accuracy	Optimal label-to-cluster matching accuracy	Higher (↑)	[0, 1]
Silhouette	Cohesion vs. separation of clusters	Higher (↑)	[-1, 1]
Davies–Bouldin Index (DBI)	Cluster overlap with nearest neighbors	Lower (↓)	[0, ∞)
Calinski–Harabasz (CH)	Separation-to-compactness ratio	Higher (↑)	[0, ∞)

Experiment 3: Acoustic pattern discovery. To assess whether the framework could move beyond cluster quality metrics and uncover potentially meaningful acoustic structures, we performed acoustic signature discovery. Recordings from seven sites across the Caribbean were embedded using the trained PAM-SimCLR encoder and clustered jointly in the shared latent space with a Gaussian Mixture Model (↑). For each cluster, mean intra-cluster cosine similarity was computed to quantify cohesion. Clusters with similarity below 0.10 were discarded, as this low threshold served only to filter out noisy or inconsistent groupings, while all remaining clusters were retained as candidate signatures. Each discovered cluster was then characterized by averaged representative spectrograms and acoustic descriptors (frequency band, bandwidth, harmonicity), which were compiled into a preliminary dictionary of acoustic signatures. Clusters were reviewed by a domain expert acoustician for potential interpretation and exclusion of clusters dominated by noise-like or low-SNR segments.

### Implementation details

All experiments were implemented in Python 3.11 using PyTorch 2.1 for deep learning models and scikit-learn 1.3 for conventional clustering algorithms. Audio preprocessing and feature extraction were performed with torchaudio 2.1, and visualization utilities used plotly 5.17 and matplotlib 3.8. Experiments were executed on a workstation equipped with an NVIDIA RTX A6000 GPU (48 GB VRAM), an AMD EPYC 7543 CPU, and 256 GB of system RAM, running Ubuntu 22.04. All numerical experiments were conducted in a reproducible environment with deterministic PyTorch settings where applicable. Reported results are averages over three runs with different random seeds. Seed-to-seed variability was limited and did not affect qualitative conclusions. For all baseline models and clustering methods, detailed architectural implementation details and hyperparameters are given in supplementary S2 Appendix.

## Results

### Validation on standard acoustic benchmarks

Before applying our framework to marine PAM, we first validated the PAM-SimCLR encoder on two widely used, label-rich datasets (BirdSet-NES [34] and UrbanSound8K [35]). These controlled, high-SNR settings provide an introductory reference point for evaluating embedding quality under favorable acoustic conditions. Importantly, this benchmark is not intended to compare alternative SimCLR variants, and all SimCLR-specific ablations for the reef PAM domain are reported separately in Table 6.

Here, we instead include a classical non-learned baseline (MFCC+K-means) to establish a consistent, domain-agnostic reference point across datasets with different annotation quality and acoustic structure. Using identical preprocessing and clustering procedures as described in Dataset and Preprocessing and Feature Extraction Approaches sections, we report internal clustering metrics including Silhouette, Davies–Bouldin Index (DBI), and Calinski–Harabasz (CH).

As summarized in Table 4, the PAM-SimCLR encoder substantially outperformed the MFCC+K-means baseline on both datasets, yielding higher Silhouette values (≥0.49), lower DBI, and higher CH scores. These results confirm that the learned representation forms compact, well-separated clusters in curated, label-rich datasets, and provide a useful contrast with the more challenging reef PAM setting examined in the remainder of this paper.

**Table 4:** Internal clustering metrics on classical acoustic datasets. Higher Silhouette and CH, and lower DBI indicate better clustering quality. This table provides reference clustering performance on clean, label-rich audio datasets; comparisons against learnable baselines for the reef PAM experiments are reported separately in Table 5.

Dataset	Method	Silhouette	DBI	CH
BirdSet-NES	MFCC + KMeans	0.34	1.75	289.70
BirdSet-NES	PAM-SimCLR	**0.49**	**1.02**	**9884.68**
UrbanSound8K	MFCC + KMeans	0.09	3.48	131.29
UrbanSound8K	PAM-SimCLR	**0.50**	**1.13**	**396.47**

**Table 5:** Evaluation of baseline methods. External metrics (ARI, AMI, Hungarian Accuracy) assess agreement with FADAR labels. Internal metrics (Silhouette, DBI, CH) assess cohesion and separability. Higher is better except for DBI.

Method	ARI	AMI	Hung.	Silhouette	DBI	CH
GTCC + MFCC	0.089	0.140	0.333	0.114	2.020	422.0
Log-MEL + PCA	0.040	0.059	0.257	0.116	1.775	14,378
VAE + GMM	0.061	0.117	0.294	0.074	3.338	914.4

**Table 6:** Evaluation of contrastive learning methods. External metrics (ARI, AMI, Hungarian Accuracy) assess agreement with FADAR labels. Internal metrics (Silhouette, DBI, CH) assess cohesion and separability. Higher is better except for DBI.

Method	ARI	AMI	Hung.	Silhouette	DBI	CH
Vanilla SimCLR	0.042	0.182	0.091	0.137	1.926	3,899
PAM-SimCLR	0.070	0.121	0.317	0.220	1.279	16,200
Semi-Supervised SimCLR	0.095	0.126	0.297	0.163	1.670	10,926
CNN-SupCon	**0.372**	**0.396**	**0.646**	**0.302**	**1.185**	**29,187**

### Evaluation of embedding quality and clustering

We next evaluated the quality of embeddings across all feature extraction methods using both external and internal metrics, and compared clustering algorithms for our best-performing representation (PAM-SimCLR). External metrics (ARI, AMI, Hungarian Accuracy) measured agreement with six predefined FADAR classes, while internal metrics (Silhouette, Davies–Bouldin Index, Calinski–Harabasz Score) assessed cohesion and separability without labels.

Embedding evaluation. Tables 5 and 6 report results for all methods using the parameters listed in Table 2. Supervised contrastive learning (CNN–SupCon) achieved the highest label agreement (ARI = 0.372, AMI = 0.396, Hungarian = 0.646) and strong internal clustering metrics. However, qualitative inspection suggested that SupCon tends to group acoustically distinct signals whenever they share a label, compressing within-class variation. In contrast, the unsupervised PAM-SimCLR model produced lower label agreement (Hungarian = 0.317) but yielded the strongest intrinsic structure (Silhouette = 0.220, DBI = 1.279, CH = 16,200), forming compact clusters that captured potentially meaningful acoustic patterns beyond the predefined categories. This interpretation is supported by quantitative within-cluster cosine similarity analysis: SupCon clusters exhibited lower similarity (0.0989) than PAM-SimCLR (0.2144), indicating that the supervised model aggregates more heterogeneous acoustic signals within each class.

The vanilla SimCLR baseline, trained with identical preprocessing, augmentations, and optimization but without the PAM-specific enhancements, exhibited weaker intrinsic clustering structure and reduced within-cluster cohesion (Silhouette = 0.137, DBI = 1.926). Consistent with this, its very low Hungarian accuracy (Hungarian = 0.091) reflects a known limitation of label-matching metrics in unsupervised settings, as the baseline objective does not encourage alignment with predefined categories and may instead organize samples according to low-level acoustic similarity.

Cepstral features (GTCC+MFCC) moderately aligned with labels (Hungarian = 0.333) but showed the weakest internal structure (Silhouette = 0.114, DBI = 2.020). VAE–GMM achieved similar label alignment to PAM-SimCLR (Hungarian = 0.294) but the lowest Silhouette score (0.074), indicating limited intra-cluster organization.

**Fig 5:**
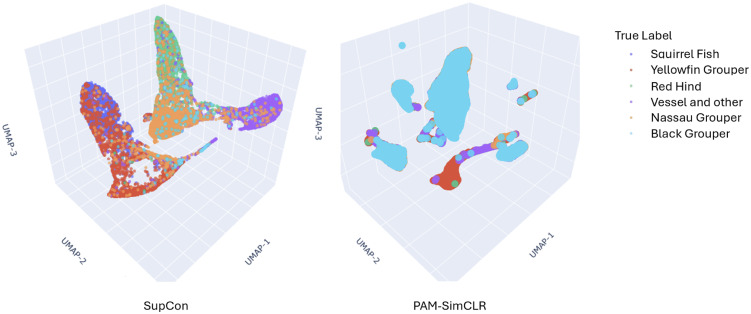
3D UMAP visualization of the test-set embeddings for (A) the supervised SupCon model and (B) the PAM-SimCLR model, colored by the six FADAR species-level labels.

To visualize these differences in embedding structure, Fig 5 shows 3D UMAP projections of the test set for both SupCon and the PAM-SimCLR model, colored by the six coarse FADAR classes. As expected for a supervised objective, the SupCon embedding forms broad, label-homogeneous regions that reflect the species boundaries. However, the quantitative results show low within-cluster cosine similarity (0.0989), and the UMAP projection is consistent with this pattern, showing substantial dispersion within each labeled region. In contrast, the PAM-SimCLR embedding produces multiple smaller and more clearly delineated regions, aligning with its higher within-cluster similarity (0.2144) and stronger intrinsic structure metrics. Together, these quantitative and qualitative results help demonstrate why PAM-SimCLR shows lower label agreement despite forming more coherent acoustic clusters. The unsupervised PAM-SimCLR preserves distinctions not captured by species-level labels, while the supervised SupCon enforces coarse class boundaries at the cost of merging acoustically heterogeneous signals.

We note that the absolute clustering scores across all methods are lower than those reported on cleaner bioacoustic and environmental sound benchmarks such as BirdSet-NES and UrbanSound8K (Table 4). This difference is expected and reflects common characteristics of real-world passive acoustic monitoring data, including lower SNR, overlapping events, higher event density, lower stereotypy of sound signals, and preprocessing steps specific to continuous long-term recordings. These challenges are not unique to marine systems and do not indicate a limitation of the representation-learning framework itself. Rather, they highlight that relative differences between methods are more meaningful than absolute score magnitudes when evaluating embedding quality under field conditions.

Clustering algorithm comparison. To assess the effect of clustering choice on the PAM-SimCLR embedding space, we compared six algorithms in Table 7. GMM and K-Means performed comparably across Silhouette and CH metrics. This suggests that the PAM-SimCLR embedding contains roughly convex or ellipsoidal clusters, which align well with the geometric assumptions of both algorithms. Density-based methods (DBSCAN, HDBSCAN) achieved high internal scores but returned only 2–3 clusters, reflecting the mostly continuous density structure of the PAM-SimCLR embedding manifold rather than a limitation of the algorithms. Spectral Clustering performed moderately but did not exceed the performance of GMM or K-Means. Given the small differences between methods, K-Means provides a competitive and computationally efficient choice, with the marginal improvements offered by GMM not clearly outweighing its added complexity.

**Table 7:** Silhouette and Calinski–Harabasz (CH) scores for different clustering algorithms applied to PAM-SimCLR embeddings at *k* = 60. Higher values indicate better clustering performance.

Clustering Method*	Silhouette (↑)	Calinski–Harabasz (↑)
KMeans	0.220	1607.2
Agglomerative	0.186	1432.8
DBSCAN	0.597	292.2
HDBSCAN	0.488	3296.6
Spectral	0.151	1209.1
GMM	0.222	1625.1

* Density-based methods (DBSCAN, HDBSCAN) returned only 2–3 clusters.

**Fig 6:**
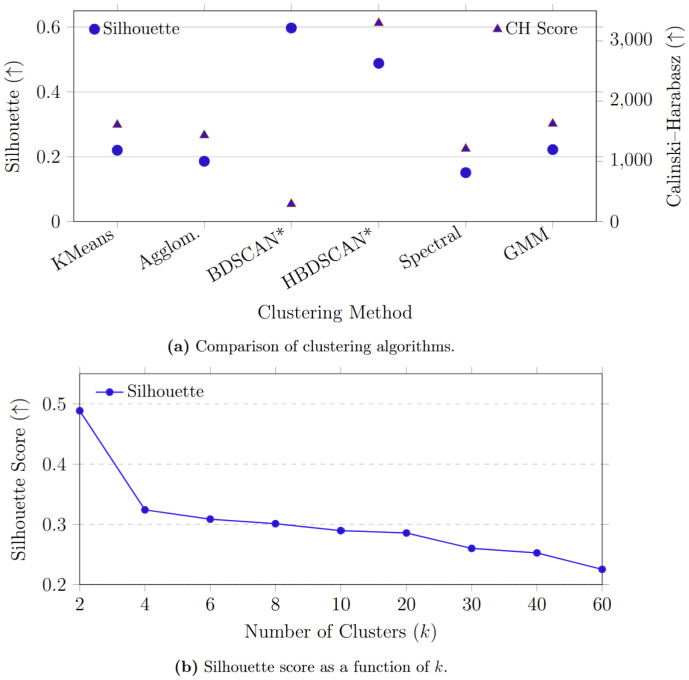
Silhouette score vs. cluster number (*k*) on PAM-SimCLR embeddings, showing decreasing cohesion at higher *k.*

Regarding the number of clusters, Fig 6 shows that increasing [0,∞ leads to a gradual decline in Silhouette score, reflecting the expected reduction in cohesion as granularity increases. Importantly, this trend should not be interpreted as evidence of ecological substructure. Low values of k=60  (e.g., ≥0.49 ) produce highly separable partitions, such as noise versus biological sounds, yet miss finer acoustic distinctions. The gradual decrease in Silhouette, rather than a sharp collapse, indicates that the embedding space can accommodate moderately higher-resolution partitions without immediate degradation of cluster quality.

### Acoustic pattern discovery

Based on the clustering comparison presented earlier, which showed that GMM and k-means behave similarly on the PAM-SimCLR embeddings, we used the PAM-SimCLR encoder paired with GMM clustering to identify recurring acoustic patterns across all reef sites. Clusters exceeding a cohesion threshold (mean within-cluster cosine similarity ↑) were retained for analysis. These clusters represent groups of events with consistent spectro–temporal structure and were summarized using averaged spectrograms and simple acoustic descriptors. The resulting collection constitutes a preliminary dictionary of recurrent acoustic patterns observed across the Caribbean datasets.

To ensure that these clusters reflected consistent acoustic structure rather than artifacts of noise level or SNR, we evaluated each retained cluster by inspecting its average spectrogram and several representative events closest to the cluster centroid. Clusters that did not exhibit coherent time–frequency structure (e.g., dominated by noise-like or low-SNR segments) were excluded. This filtering step ensures that the patterns presented reflect meaningful spectro–temporal similarity.

**Fig 7:**
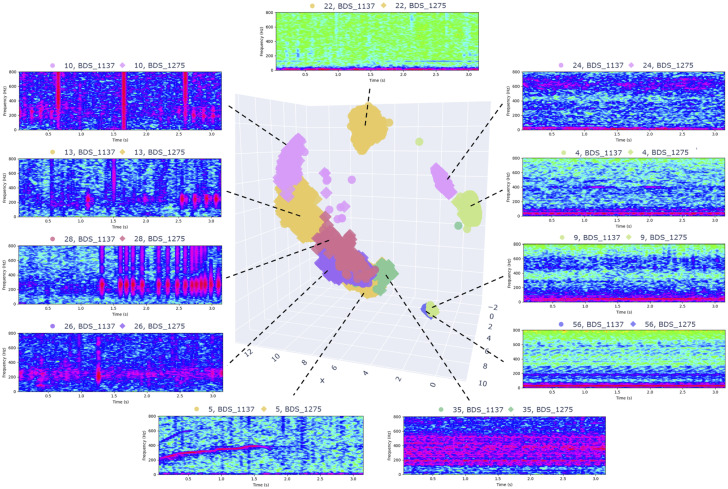
UMAP projection of Puerto Rico BDS latent space (PAM-SimCLR embeddings), showing the 11 acoustic signatures present after applying the cohesion threshold. Points are colored by cluster ID and number out of the original 60 clusters created.

**Fig 8:**
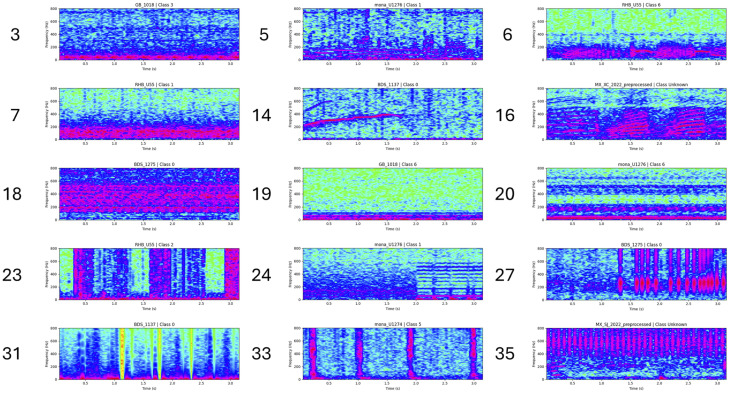
Spectrogram gallery of representative acoustic signatures identified through clustering across all sites. The examples include recurrent sounds associated with spawning species observed at multiple locations, as well as sound signatures restricted to particular sites. Distinct anthropogenic sounds, such as vessel noise, also appear as site-specific signatures. The full spectrogram gallery is provided in S1 Appendix. Sounds with similar spectro-temporal structure may be grouped into the same cluster, even when visual differences are subtle (e.g., Clusters 3 and 5).

The spatial organization of the clusters in the learned embedding space is shown in Fig 7, where a UMAP projection of the Puerto Rico BDS dataset displays the 11 GMM-derived clusters used for this site after the threshold was applied. Colors correspond directly to cluster IDs out of the 60 clusters formed, which are distinct from the IDs given in the dictionary of the full dataset. Quantitative summaries of the number of acoustic patterns and their mean cohesion across sites are reported in Table 8. Representative examples from the resulting acoustic pattern dictionary are provided in Table 9 and Fig 8, illustrating both widely occurring and site-specific spectro–temporal structures.

**Table 8:** Number of acoustic signatures discovered across 10 sites. Cohesion is reported as mean intra-cluster cosine similarity.

Site	Acoustic Signatures	Mean Cohesion
Puerto Rico BDS	11	0.1199
St Thomas GB	13	0.2128
St Thomas RBH	9	0.4058
Mona Elbow	16	0.2030
Mona H6	11	0.1572
Puerto Rico ALS	11	0.2287
Puerto Rico ALS Deep	14	0.1184
Mexico San Juan	8	0.1785
Mexico Punta Allen	4	0.1068
Mexico Xcalak	4	0.2259

**Table 9:** Example entries from the acoustic signature dictionary.

Cluster ID	Sites	Freq Band (Hz)	Interpretation of potential pattern structure
3	GB, Mona Elbow, Mona H6, ALS Deep, ALS, MX XC, MX PA	50–100	Large ship (may include black grouper)
5	GB, Mona Elbow, ALS Deep, ALS	0–200	Yellowfin grouper and red hind (RH2)
6	RBH	200–800	Red hind (RH1)
7	RBH, Mona H6, ALS	0–200	red hind chorus
14	BDS	200–600	Marine mammal
16	MX XC, MX PA	0–600	Toadfish
18	BDS, Mona Elbow, Mona H6, ALS Deep, ALS	100–600	Vessel noise 4
19	GB, RBH, BDS, ALS, MX SJ	0–100	Vessel noise 5
20	BDS, Mona Elbow, Mona H6, ALS	0–200, 400–600	Vessel noise 6 (only in Puerto Rico)
24	Mona Elbow	0–600	Vessel noise 9
27	BDS, ALS Deep, Mona Elbow, Mona H6	200–800	Biotic unknown
35	MX SJ	400–800	Vessel noise 13

Taken together, these results indicate that the unsupervised framework can organize long-term reef recordings into stable and coherent spectro–temporal patterns that are not captured by predefined class labels. The representative examples show that the retained clusters contain consistent acoustic structure rather than noise- or SNR-driven artifacts, and that some patterns appear broadly across sites while others are localized. While ecological validation requires species-annotated data and is beyond the scope of this study, these results demonstrate that the learned representation supports the unsupervised grouping of recurrent acoustic events and provides a foundation for constructing a scalable acoustic pattern dictionary for Caribbean PAM datasets.

## Discussion

### Machine learning impact

Unsupervised clustering in PAM remains difficult due to overlapping calls, heterogeneous environments, and subtle across-species variation [36]. Our results reproduce patterns reported in prior ecoacoustic studies, including the sensitivity of cepstral features to noise and habitat variability [10,37,38]. Cepstral features such as MFCCs and GTCCs capture broad spectral differences but tend to fragment into unstable clusters under noisy background conditions [37,38]. Autoencoder-based pipelines, including VAEs and deep embedded clustering with GMMs, have shown promise for stereotyped calls in birds and marine mammals [20,21,39], yet our results suggest that VAE + GMM approaches may struggle to generalize to diverse reef soundscapes. The VAE + GMM method achieved a silhouette score of lower than 0.10 on PAM data, indicating very weakly defined clusters and boundaries. This limitation reflects a core assumption of VAE + GMM approaches, that latent clusters are approximately Gaussian and calls are stereotyped, which breaks down in low-SNR, overlapping, and highly variable reef environments.

The supervised embedding space aligned closely with known labels, consistent with prior bioacoustic monitoring studies [40,41]. However, while maximizing classification accuracy, the supervised objective collapsed distinct sound types with similar spectral patterns into single classes, obscuring potentially ecologically relevant variations. This trade-off between label accuracy and discovery is often overlooked but is central to representing real-world soundscapes.

Our PAM-adapted contrastive learning pipeline offers an alternative to reconstruction-based unsupervised approaches for representing complex marine soundscapes. By tailoring augmentations to the characteristics of underwater acoustics, including event-centric cropping, frequency masking, and teacher-guided multi-positive pairing, the learned embeddings preserved variability among sound types while maintaining robustness to noise and overlapping signals. In contrast to GMVAE-style approaches, which rely primarily on reconstruction error to structure latent space and may struggle to separate acoustically similar events [39], contrastive objectives impose relational constraints that encourage separation between distinct sound patterns without collapsing within-pattern variability.

Similar advantages of self-supervised representations have been reported in avian and general audio domains, where learned embeddings captured temporal and site-level variation beyond predefined species labels [10,25]. In our study, applying this framework at scale yielded stable clustering structure across hundreds of thousands of reef recordings and facilitated the identification of 33 candidate acoustic signatures in the 0–800 Hz band. Together, these findings suggest that contrastive learning provides a practical, label-efficient basis for exploratory and large-scale ecoacoustic analysis.

### Validation of regional and site-level characteristics

We provide here some examples of how some of the identified clusters reflect the underlying soundscape characteristics of the sites chosen in this study. Across sites, several characteristics emerged from clustering. In particular, Puerto Rico exhibited the greatest acoustic diversity, with 15 distinct signatures that span grouper calls, unknown pulses and pulse trains, low-frequency calls from marine mammals identified as Humpback whales [26], and numerous vessel sounds. The coexistence of diverse biological and anthropogenic signatures indicates that human-generated noise is a prominent component of these soundscapes. This result is confirmed by the study of [42]. They found that the soundscapes were significantly different between the northern Antilles, the Windward and the Leeward Islands. The northern and Windward Islands soundscape was dominated by ship traffic and Humpback whale song that occurred on 49–93% of recording days. Prior studies have shown that reefs near human population centers can experience acoustic masking and altered call dynamics [43]. Future unsupervised pattern-analysis studies could examine co-occurrence relationships between specific anthropogenic and biological sound types to explore whether these interactions relate to masking phenomena reported in the literature.

One striking example was the RHB off St. Thomas, US Virgin Islands. RHB is a documented spawning aggregation site mainly for red hind [44], and its recordings were characterized by unusually clean chorus periods (Cluster 7 as shown in Fig 8 and Table 9) and call types such as RH1 and RH2 (Cluster 6&5, respectively) associated with courtship [7,8,45]. Accordingly, it confirms that RHB is a species specific site where a single species aggregate to spawn. This acoustic clarity could be due to the lack of individual overlapping sounds produced by other organisms in the red hind call frequency range. These properties, thus, could make RHB valuable as a low-noise control to separate biological signatures from anthropogenic noise and to benchmark cluster cohesion. Cluster 7 was found only in non Nassau grouper spawning sites (RBH, Mona H6, ALS), all exhibiting red hind choruses in the 0–200 Hz band. This consistent separation suggests predictive acoustic markers of species specific spawning sites that could complement diver surveys and fisheries monitoring by providing scalable, non-invasive classification of marine habitats [8].

The three Caribbean Mexico sites showed sharp contrasts. Xcalak and Punta Allen were dominated by toadfish tonal harmonic sequences (0–600 Hz, Cluster 16), absent elsewhere [46,47]. This site-restricted dominance highlights the framework‘s ability to isolate species-specific activity. By contrast, San Juan exhibited almost no consistent biological clustering, which may reflect reduced fish abundance or poor sound transmission. These differences underscore the value of unsupervised approaches for detecting soundscape characteristics across neighboring habitats. At Puerto Rico‘s BDS site, strong low-frequency marine mammal calls (200–600 Hz, Cluster 14) were detected — unique among all sites. This demonstrates sensitivity not only to fish but also to wider ecosystem contributors such as marine mammals, supporting recent calls for multi-taxa acoustic indicators [8,48].

Cross-site patterns also emerged. For example, Cluster 3 appeared in recordings from multiple regions, suggesting a widely shared anthropogenic source such as a shipping route. In contrast, Cluster 35 was observed only at San Juan, indicating a site-specific sound type. More broadly, 23 clusters occurred across multiple sites, while 10 clusters were restricted to a single location (see S1 Appendix). These observations show that the clustering produces a mixture of cross-site and site-specific acoustic patterns

### Limitations and future work

Several limitations remain. First, our study focused on Caribbean reefs; transferability to Pacific, temperate, or deep-sea systems has not been tested. Second, although PAM-SimCLR produced internally coherent clusters, ecological interpretation requires validation through in situ observations and behavioral studies [7,36,48]. Separating calls of similar patterns, for example red hind RH2 and yellowfin tonal call appeared to be a challenge and were part of the same clusters. A similar challenge was observed for the separation of low-frequency ship sound and black grouper calls, underscoring the need for larger, more balanced datasets and potentially hierarchical contrastive learning. This may also be the fault of augmentations, which help separate dissimilar calls and patterns but may actually encourage clustering when differences are too subtle. Emerging frameworks involving diffusion-driven contrastive learning may provide more granular latent organization, potentially improving separation of acoustically similar subtle call variations.

Methodologically, we restricted our analysis to the 0–800 Hz band associated with grouper calls. This was intentional given our focus on spawning aggregation monitoring but likely excluded higher-frequency signals of ecological importance. In addition, all recordings were downsampled to 10 kHz and segmented into 2-second windows. While appropriate for low- to mid-frequency fish calls and vessel noise, these settings limit applicability to higher-frequency taxa such as snapping shrimp or dolphins. Adapting the framework to such species would require retaining higher sampling rates and using different temporal windows. We also balanced the dataset using predictions from the FADAR grouper classifier, which may have filtered out faint or unrecognized calls. This was deemed necessary for handling a large unlabeled dataset but represents a potential source of bias.

Future work should explore transformer-based audio models, including both speech-derived architectures [22] and recent bioacoustic foundation models. NatureLM-audio [49] represents a large-scale audio–language foundation model tailored for bioacoustics and has demonstrated strong zero-shot generalization across unseen taxa. Species-specific SSL models such as Dolph2Vec [50] further illustrate how transformer-based encoders can capture fine-grained structure within a particular vocal repertoire. While these models show impressive cross-domain and within-species performance, their computational demands and reliance on curated, pre-segmented corpora make them challenging to deploy directly on continuous reef PAM data. Additional directions include augmentations that account for diel cycles or snapping-shrimp interference, and semi-supervised fine-tuning with limited expert labels to better connect unsupervised discovery with species-level classification.

## Conclusion

This study shows that contrastive learning provides a practical framework for representing complex marine soundscapes without dense annotation. Whereas supervised pipelines align closely with predefined labels and conventional unsupervised approaches often suffer from instability, contrastive objectives can balance robustness and acoustic variability when adapted to passive acoustic monitoring data. Using acoustically informed augmentations and multi-positive pairing, the learned embeddings supported consistent unsupervised organization of large-scale reef recordings across sites. The resulting representations capture recurring sound patterns, distinguish site-shared and site-specific signatures, and support the construction of preliminary acoustic dictionaries without reliance on species-level labels. Together, these findings suggest that contrastive learning offers a scalable, discovery-oriented approach for exploratory ecoacoustic analysis and a basis for future ecological validation and targeted monitoring in complex marine environments.

## Supporting information

S1 Appendix Acoustic signatures. This appendix summarizes the principal acoustic signatures identified during passive acoustic monitoring (PAM) surveys, grouped into three categories: (1) Signature species, (2) Unknown biotic sounds, and (3) Ambient noise and vessel sounds. Each table provides signature ID, frequency band, site occurrence, and descriptive notes, while Fig A, Fig B, and Fig C in S1 Appendix illustrate representative spectrograms for each group.
(PDF)

S2 Appendix Method details. This appendix provides complete architectural, preprocessing, and training specifications for all baseline and proposed models, including detailed loss formulations, clustering configurations, and hyperparameters.
(PDF)
