# Supplementary material for: Contrastive learning for passive acoustic monitoring: A framework for sound source discovery and cross-site comparison in marine soundscapes
Source: PLoS Comput Biol. 2026 Mar 6;22(3):e1014005. doi: 10.1371/journal.pcbi.1014005 (PMC12978570; doi:10.1371/journal.pcbi.1014005)
Supplement: S1 Appendix — This appendix summarizes the principal acoustic signatures identified during passive acoustic monitoring (PAM) surveys, grouped into three categories: (1) Signature species, (2) Unknown biotic sounds, and (3) Ambient noise and vessel sounds. Each table provides signature ID, frequency band, site occurrence, and descriptive notes, while Fig A, Fig B, and Fig C in S1 Appendix illustrate representative spectrograms for each group. (PDF) [file pcbi.1014005.s001.pdf]

Appendix S1: Acoustic Signatures

This appendix summarizes the principal acoustic signatures identified during passive acoustic monitoring (PAM) surveys. To aid interpretation, the signatures are grouped into three broad categories according to their dominant spectral and temporal characteristics: biotic and anthropogenic tones, vessel sounds, and biotic and anthropogenic bursts and pulse trains sounds. Each table provides the signature ID, frequency range, observed sites, and descriptive notes, while accompanying figures illustrate representative spectrograms. Together, these tables and figures serve as a reference "dictionary" of recurring patterns that can be used to compare across sites, years, and ecological contexts.

Category 1 — Signature species

Sig ID	Sites	Freq Band (Hz)	Notes
5	GB, Mona Elbow, ALS Deep, ALS	0–200	Yellowfin grouper and red hind (RH2)
6	RHB	200–800	Red hind (RH1)
7	RHB, Mona H6, ALS	0–200	Red hind chorus
9	GB, Mona Elbow, Mona H6, ALS Deep, MX SJ	150–300	Nassau Grouper (N2)
10	BDS, Mona H6, MX SJ, MX PA	200–400	Nassau Grouper (N1)
14	BDS	200–600	Marine mammal
16	MX XC, MX PA	0–600	Toad fish

Category 2 — Unknown biotic sounds

Sig ID	Sites	Freq Band (Hz)	Notes
8	Mona Elbow	200–400	Biotic unknown
11	RHB, BDS	200–400	Biotic unknown
13	GB, ALS Deep, Mona Elbow, MX XC, MX PA	200–400	Biotic unknown
27	BDS, ALS Deep, Mona Elbow, Mona H6	200–800	Biotic unknown
28	GB, MX SJ	200–800	Biotic Unknown
29	RHB	200–800	Biotic unknown
34	GB, Mona H6, ALS Deep	Full (0–800)	Biotic unknown

Category 3 — Ambient noise and vessel sounds

Sig ID	Sites	Freq Band (Hz)	Notes
1	RHB, GB, Mona Elbow, ALS Deep	25–75	Large ship
2	RHB, GB, Mona Elbow, Mona H6, ALS Deep	0–75	Large ship with high background noise
3	GB, Mona Elbow, Mona H6, ALS Deep, ALS, MX XC, MX PA	50–100	Large ship
4	BDS, ALS, MX SJ	0–50	Large ship with masking noise

Sig ID	Sites	Freq Band (Hz)	Notes
12	RHB, ALS Deep	0–400	Vessel noise 1
15	GB, ALS Deep, ALS, MX XC, MX SJ	400–600	Vessel noise 2
17	BDS, Mona Elbow, Mona H6	600–800	Vessel noise 3
18	BDS, Mona Elbow, Mona H6, ALS Deep, ALS	100–600	Vessel noise 4 (only in Puerto Rico)
19	GB, RHB, BDS, ALS, MX SJ	0–100	Vessel noise 5 (Common across biogeographies)
20	BDS, Mona Elbow, Mona H6, ALS	0–200, 400–600	Vessel noise 6 (only in Puerto Rico)
21	BDS, Mona Elbow, Mona H6, ALS Deep, ALS	Full (0–800)	Vessel noise 7 (only in Puerto Rico)
22	RHB, GB, ALS, ALS Deep	Full (0–800)	Vessel noise 8 (only in Puerto Rico and St Thomas)
24	Mona Elbow	0–600	Vessel noise 9
25	Mona Elbow	0–200	Vessel noise 10
26	GB	0–200	Vessel noise 11
30	Mona Elbow	Full (0–400)	Vessel noise 12
32	GB, BDS, ALS, ALS Deep, Mona Elbow	Full (0–800)	Instrument noise
35	MX SJ	400–800	Vessel noise 13
36	MX SJ	Full (0–800)	Anthropogenic sound in high background noise

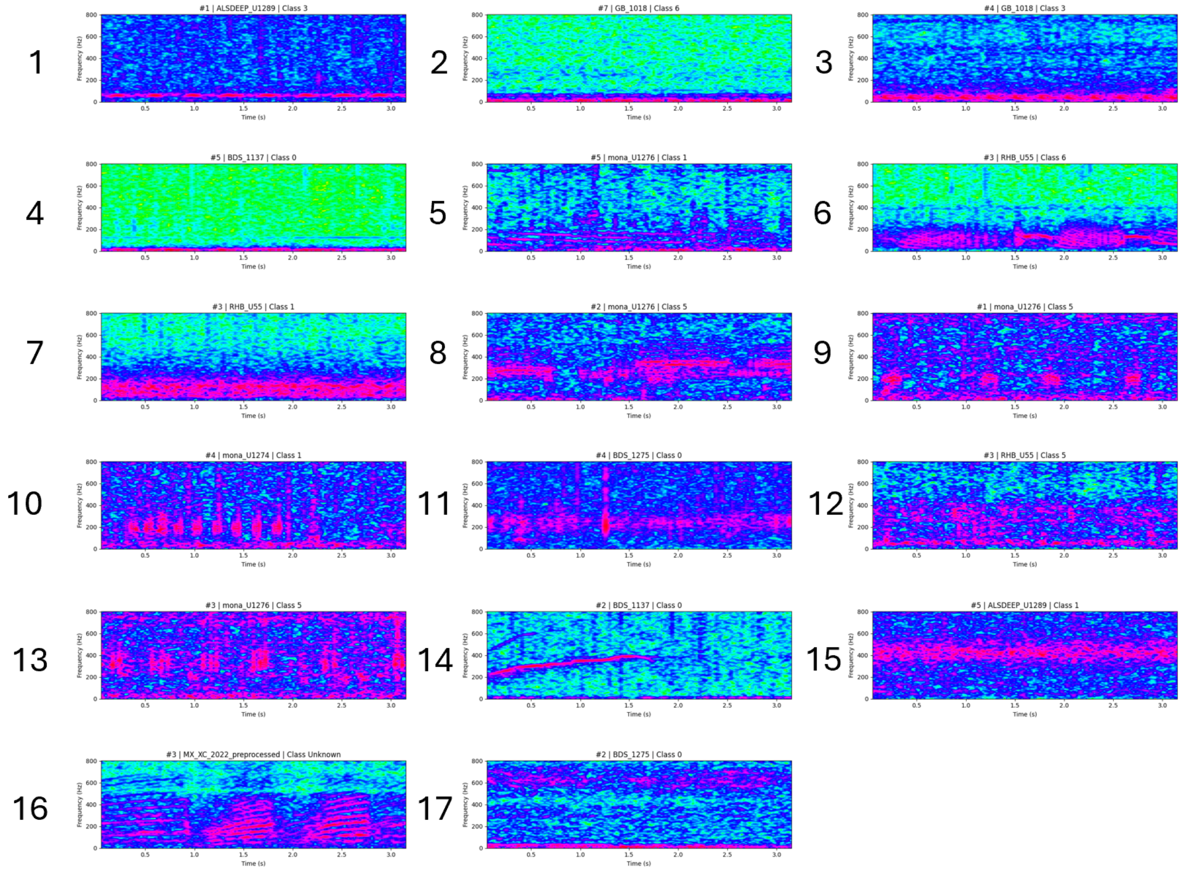

Figure A: Representative spectrograms of spawning species.

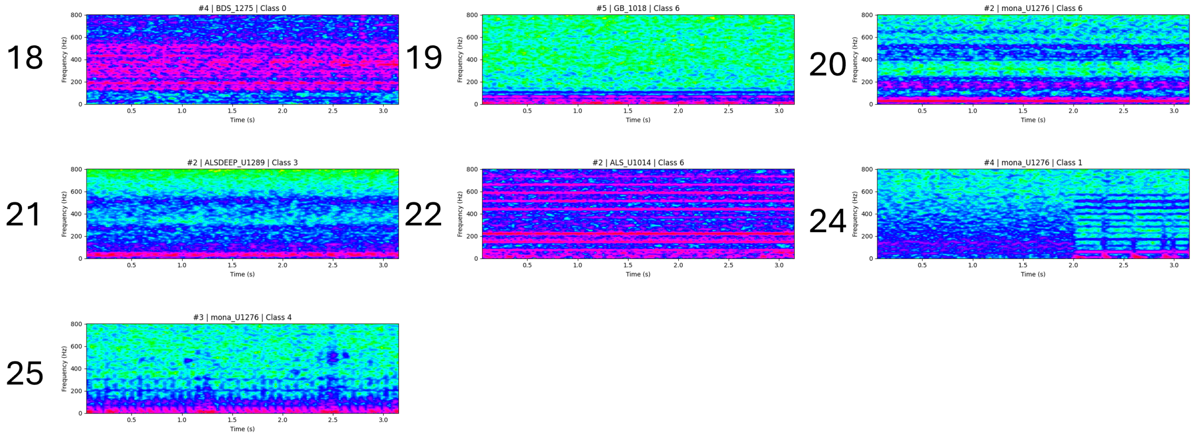

Figure B: Representative spectrograms of unknown biotic sounds.

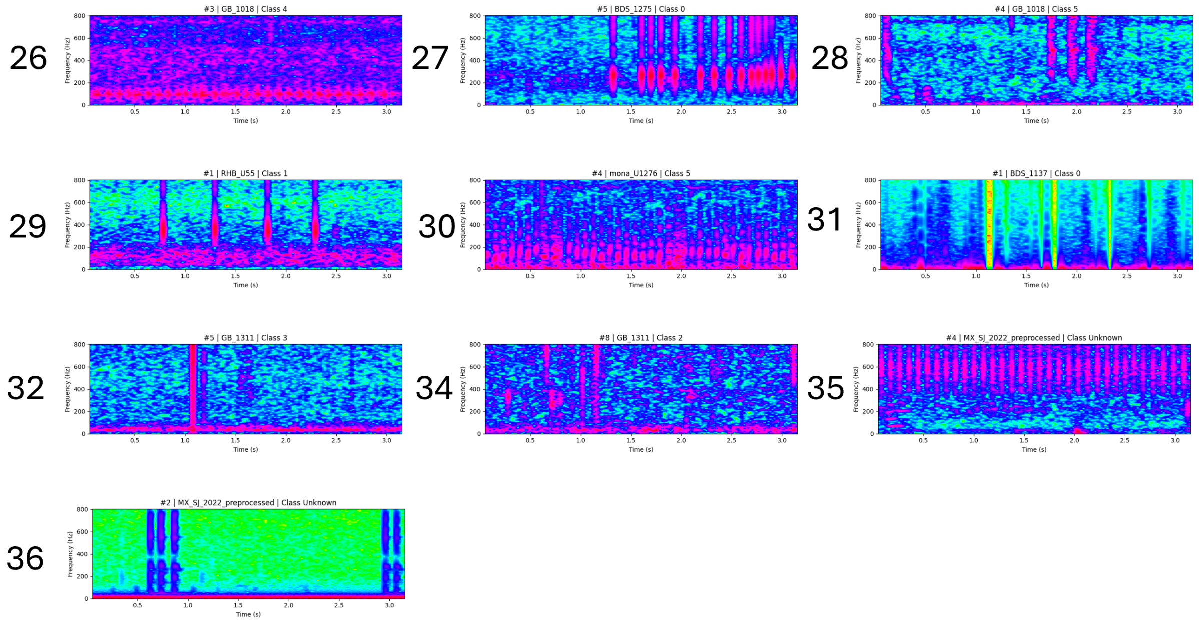

Figure C: Representative spectrograms of anthropogenic sounds.
