# Supplementary material for: Contrastive learning for passive acoustic monitoring: A framework for sound source discovery and cross-site comparison in marine soundscapes
Source: PLoS Comput Biol. 2026 Mar 6;22(3):e1014005. doi: 10.1371/journal.pcbi.1014005 (PMC12978570; doi:10.1371/journal.pcbi.1014005)
Supplement: S2 Appendix — This appendix provides complete architectural, preprocessing, and training specifications for all baseline and proposed models, including detailed loss formulations, clustering configurations, and hyperparameters. (PDF) [file pcbi.1014005.s002.pdf]

Appendix S2: Method Details

This supplementary document provides expanded architectural, preprocessing, and training specifications for all baseline and proposed models. All hyperparameters were tuned using validation data and are reported here for reproducibility.

A. Datasets and Preprocessing

Waveform handling. All audio is converted to mono and amplitude-normalized to $[-1, 1]$. Unless otherwise specified, we resample to 10 kHz for reef PAM experiments.

Spectrograms. We compute log-Mel spectrograms with the following default parameters unless otherwise stated: FFT size 1024, hop 512, and either 64 or 128 Mel bands in the 0–5 kHz range. Log compression uses $\log(\max(x, 10^{-5}))$; per-sample standardization is applied when noted.

Event-centric multi-crop. For self-supervised models, each training sample yields **four** crops: **two global** (256 frames) and **two local** (96 frames) views centered on energy peaks with ± 24 frame jitter. Crops are optionally resized to a fixed canvas (e.g., 128×256) for stable batch statistics.

B. Model Families (Summary Tables)

C. Full Objective Function

The training objective combines contrastive alignment, local–global invariance, and variance regularization under a teacher–student framework with a feature memory bank:

$$\mathcal{L}_{\text{total}} = \alpha\,\mathcal{L}_{\text{ctr}} + \beta\,\mathcal{L}_{\text{siam}} + \gamma\,\mathcal{L}_{\text{vic}}, \tag{1}$$

with weights α, β, and γ.

(1) Teacher-guided multi-positive contrastive loss

For a batch of B audio samples with **two global** crops each, let $z_g^{(1)}, z_g^{(2)} \in R^d$ be online projections and $t_g^{(1)}, t_g^{(2)}$ their teacher counterparts. A FIFO bank of M teacher vectors augments negatives. The loss is

$$\mathcal{L}_{\text{ctr}} = -\frac{1}{2B} \sum_{i=1}^{2B} \sum_{j=1}^{2B+M} W_{ij}^{\text{full}} \log \frac{\exp(\text{sim}(z_i, z_j)/\tau)}{\sum_{l=1}^{2B+M} \exp(\text{sim}(z_i, z_l)/\tau)}, \tag{2}$$

where sim is cosine similarity and τ is temperature. The teacher-derived weights are

$$W_{ij} = \begin{cases} 1, & \text{paired augmentation of } i, \\ \dfrac{1}{Z_i} \max\left(0, \dfrac{1 + \text{sim}(t_i, t_j)}{2}\right), & \text{sim}(t_i, t_j) > \theta, \\ 0, & \text{otherwise,} \end{cases} \qquad Z_i = \sum_j W_{ij}.$$

Self-pairs are masked. The fraction is the softmax probability that anchor z_i selects z_j among batch+bank candidates.

(2) SimSiam invariance (global–local)

We encourage local–global consistency via a symmetric cosine loss between the online predictor and a stop-gradient target:

$$\mathcal{L}_{\text{siam}} = -\tfrac{1}{2}\Big[\cos\angle\big(p_g^{(1)},\, \text{sg}(z_\ell)\big) + \cos\angle\big(p_\ell,\, \text{sg}(z_g^{(1)})\big)\Big], \tag{3}$$

where $p_g^{(1)}$ and p_ℓ are predictor outputs for a global and local view, respectively. If locals are unavailable, we fallback to (global, global).

(3) VICReg

We apply variance–invariance–covariance regularization to the same pair (z_a, z_b):

$$\mathcal{L}_{\text{vic}} = \lambda_{\text{sim}}\|z_a - z_b\|_2^2 + \lambda_{\text{var}}\sum_d \big[\max(0, \gamma - \sigma_d(z_a)) + \max(0, \gamma - \sigma_d(z_b))\big] + \lambda_{\text{cov}}\sum_{d\neq d'} \big(C_{dd'}(z_a)^2 + C_{dd'}(z_b)^2\big), \tag{4}$$

where σ_d is per-dimension std and $C_{dd'}$ are off-diagonal covariances. The three terms promote invariance, non-collapse, and decorrelation.

(4) Feature-bank consistency

Teacher embeddings from the current mini-batch are *enqueued after the optimization step* into a FIFO bank of size M. This provides a slowly evolving negative set and avoids using an item as its own negative or positive in the same step.

D. Clustering Configuration and Metrics

Internal metrics

Silhouette:

$$s(i) = \frac{b(i) - a(i)}{\max\{a(i), b(i)\}},$$

with $a(i)$ intra-cluster mean distance and $b(i)$ nearest-cluster mean distance. **Davies–Bouldin (DBI)**:

$$\text{DBI} = \frac{1}{k}\sum_{i=1}^{k} \max_{j\neq i} \frac{\sigma_i + \sigma_j}{d(c_i, c_j)}.$$

Calinski–Harabasz (CH):

$$\text{CH} = \frac{\text{tr}(B_k)}{\text{tr}(W_k)} \cdot \frac{n-k}{k-1}.$$

External metrics

Clusters are compared to the six annotated classes using Adjusted Rand Index (ARI), Adjusted Mutual Information (AMI), and Hungarian accuracy. **ARI**:

$$\text{ARI} = \frac{\text{RI} - E[\text{RI}]}{\max(\text{RI}) - E[\text{RI}]},$$

where RI counts pairwise agreements. **AMI**:

$$\mathrm{AMI}(U,V)=\frac{\mathrm{MI}(U,V)-E[\mathrm{MI}]}{\max(H(U),H(V))-E[\mathrm{MI}]},\quad \mathrm{MI}(U,V)=\sum_{i,j}\frac{n_{ij}}{n}\log\left(\frac{n\,n_{ij}}{a_i b_j}\right).$$

Hungarian accuracy:

$$\mathrm{Acc}=\tfrac{1}{n}\max_{\pi\in S_k}\sum_{i=1}^{k}M_{i,\pi(i)}.$$

Table 1: Model configurations and training setups (summary).

Method	Input / Preprocessing	Architecture	Training Setup
**Classical (GTCC+MFCC)**	Waveform → GTCC (25 ms Hann, 10 ms hop, 128 ERB filters, log + DCT, 80 coeffs) + MFCC (STFT 1024, hop 512, 64 Mels, 13 coeffs); concat.	Feature extraction only; no trainable parameters.	Computed with `librosa` / `SciPy`; used as fixed feature vectors for clustering baselines.
**Classical (Log-Mel + PCA)**	Waveform → Log-Mel (STFT 1024, 64 Mels, hop 512, 0–5 kHz); flatten → PCA (100 comps).	No trainable parameters; PCA dimensionality reduction.	PCA fit on train only; applied to val/test.
**CNN Latent Space**	Log-Mel as above; zero-pad/trim to 224×224; 3-channel replication.	Pretrained CNN (NOAA fish-call); penultimate FC as embedding.	Inference only; ONNX with GPU backend.
**CNN-SupCon**	Single-channel Log-Mel; SpecAugment (time/freq mask).	ResNet-18; MLP head with BN–ReLU–Dropout×3.	SupCon loss (τ=0.03); Adam (2×10^{-3}, wd 10^{-4}); batch 256; mixed precision; 200ep.
**VAE + GMM**	Log-Mel (64 bands, 10 kHz); normalized.	Enc: 3 conv (ReLU) + FC → $(\mu, \log\sigma^2)$; Dec: FC → deconv (ReLU) → Sigmoid.	MSE + KL; Adam (10^{-3}); batch 256; 80:20 split; GMM (k=6) on latents.
**SimCLR (ours, teacher-guided multi-positive)**	**4 crops / sample:** 2 global (256f) + 2 local (96f). Random Mel configs: (64,1024,512), (128,1024,256), (128,2048,512). Aug: time/freq mask, spectral notch, time shift (≤12%), noise (σ=0.01), time-crop.	ResNet-18 (1-ch); projector: 512→256→128 (BN, ReLU, ℓ_2 norm). Predictor (SimSiam): 128→256→128. EMA teacher (momentum 0.99).	Losses: (1) teacher-guided multi-positive NT-Xent (τ=0.07, top-k=5, θ=0.7) + feature bank (65,536); (2) SimSiam (global↔local); (3) VICReg. AdamW (3×10^{-4}, wd 10^{-4}); warmup 10 ep + cosine; grad clip 1.0; batch 256 (accum 2); FP16/BF16; 200 ep.
**SupCon (ours)**	Same as SimCLR.	ResNet-50; 2-layer projection MLP.	Supervised contrastive; Adam (3×10^{-4}); batch 256; 200 ep.

Table 2: Key hyperparameters for the teacher-guided multi-positive SimCLR (ours).

**Component**	**Setting**
Sampling / Crops	2 global (256 frames) + 2 local (96 frames), energy-centered with $\pm$24 frame jitter; resize to 128$\times$256 (bilinear).
Mel front-ends	Random choice per view: (64 Mels, FFT 1024, hop 512), (128 Mels, 1024, 256), (128 Mels, 2048, 512).
Augmentations	Time/frequency masking (SpecAugment), *spectral notch* dropout, temporal shift ($\leq$ 12%), Gaussian noise (σ=0.01), random time-cropping.
Backbone / Heads	ResNet-18 (1-ch); projector 512$\rightarrow$256$\rightarrow$128 (BN, ReLU, ℓ_2); predictor 128$\rightarrow$256$\rightarrow$128.
EMA Teacher	Momentum m=0.99 with cosine ramp toward 0.9995; EMA buffers updated.
Contrastive	Teacher-guided multi-positive NT-Xent: temperature τ=0.07; soft positives: top-k=5, threshold θ=0.7; negatives include FIFO feature bank.
Feature Bank	FIFO queue of normalized teacher embeddings, size 8192; *enqueue-after-step* to avoid stale gradients.
Auxiliary Losses	SimSiam (global$\leftrightarrow$local); VICReg (variance, invariance, covariance).
Objective Weights	α=1.0 (ctr), β=0.1 (siam), γ=0.1 (vic).
Optimization	AdamW (3$\times$10^{-4}, wd 10^{-4}); warmup 10 epochs + cosine; batch 256 (accum 2); grad clip 1.0; mixed precision; channels-last.

Table 3: Clustering algorithms and hyperparameters.

**Algorithm**	**Parameters**
K-Means	k=6 (FADAR classes), init=`k-means++`, max_iter=300, n_init=10.
Agglomerative	k=6, linkage=Ward, affinity=Euclidean.
Spectral Clustering	k=6, affinity=10-NN graph, eigen_solver=`arpack`.
Gaussian Mixture (GMM)	Components=6, covariance=full, init=k-means, max_iter=200.
DBSCAN	ε=0.5, min_samples=10, metric=Euclidean.
HDBSCAN	min_cluster_size=30, min_samples=10, metric=Euclidean, selection=EOM.
